# Insulin resistance factors associated with differentiated thyroid carcinoma and malignant cytology: serum thyroxine as an associated factor

**DOI:** 10.1080/07853890.2025.2530697

**Published:** 2025-07-15

**Authors:** Lina Guzikauskas Celescuekci, Mariana Pereira Pinto, Ilka Mara Borges Botelho, Elizabeth João Pavin, Denise Engelbrecht Zantut-Wittmann

**Affiliations:** a Faculty of Medicine, Pontifical Catholic University of Campinas, Campinas, São Paulo, Brazil; b Division of Endocrinology, Department of Internal Medicine, Faculty of Medical Sciences, University of Campinas, Campinas, São Paulo, Brazil

**Keywords:** Cardiovascular risk, insulin sensitivity, thyroid cancer, thyroid nodules

## Abstract

**Background:**

Factors related to cardiovascular risk and insulin sensitivity seem to be related to the increase in the number of thyroid cancer (TC) diagnosis, but there is a lack of studies to corroborate these hypotheses.

**Methods:**

This observational and cross-sectional study evaluated ultrasonographic, clinical, cytological and anatomopathological characteristics of thyroid nodules (TN) related with factors indicative of cardiovascular risk (CVR), serum lipids, and glucose profiles through medical records, and interviews during routine medical consultation.

**Results:**

The study included 160 TN patients, 85.53% women, 41.3% with obesity, 24.18% with type 2 Diabetes Mellitus, 63.4% with arterial hypertension, 9.15% with previous CVR, 38.16% with dyslipidemia. 38.5% had Papillary TC, 8.5% Follicular TC. Regarding cytology categories (Bethesda,B), 34.64% BII, 8.5% BIII, 11.11% BIV, 26.8% BV, 8.5% BVI. Microcalcifications were associated with higher Framingham and ASCVD CVR Scores, fasting glucose and glycated hemoglobin. BII TN had lower fasting glucose; BIII greater glycated hemoglobin; and BIV higher both CVR scores. Framingham Score was lower in papillary TC, abdominal waist was lower in follicular TC. Higher FT4 increased 4.7 times the chance of malignant cytology. Higher HDL was an associated factor for malignant cytology (OR = 1.064) and higher FT4 for Differentiated TC (OR = 7.409).

**Conclusion:**

TN with greater malignant potential on ultrasound and cytology were associated with greater CVR and altered glucose metabolism. Larger goiters and multiple nodules were related to factors indicative of insulin resistance. Malignant cytology was related to hormonal factors that induce greater cell replication, such as thyroxine and insulin resistance.

## Introduction

Thyroid nodules are found in 65% of the general population. About 90% of the cases are benign, asymptomatic, occurring mainly in women, the elderly, individuals with iodine deficiency, and/or with a previous history of cervical exposure to external radiation [1].

There has been a significant increase in the number of thyroid cancer diagnosis, mainly because of the most often detection of subclinical thyroid tumors due to widespread use of ultrasonography and fine-needle puncture cytology (FNAC) [2]. However, the development of diagnostic techniques does not seem to explain alone the increase in the number of cases. Other factors are probably concurring, as there is an increase in the number of cases of all nodule sizes and not only those that are not palpable, as would be expected to occur with the more frequent use of diagnostic technologies [3]. Several risk factors are classically associated with differentiated thyroid carcinoma, such as a family history of thyroid cancer or hereditary syndromes that include medullary thyroid cancer, especially the type 2 multiple endocrine neoplasm, history of personal exposure to external radiation in childhood or adolescence, rapid nodular growth, and/or hoarseness [1]. Nevertheless, factors related to cardiovascular risk and insulin sensitivity, such as greater waist circumference, high concentrations of triglycerides and glycated hemoglobin (HbA1c), and high Homeostasis Model Assessment-Insulin Resistance Index (HOMA-IR), seem to have a relationship with the presence of nodules [4,5], but there is still a lack of studies to corroborate these hypotheses.

The present study aims to assess the relationship between factors related to cardiovascular risk with thyroid nodules biochemical and ultrasound characteristics, cytological, and pathological autoimmunity determinants.

## Methods

### Study design

An observational and cross-sectional analytical study was carried out, involving patients over 18 years of age, of both sexes, followed at the Thyroid Nodule Service of the Endocrinology Division of a tertiary Hospital. Thyroid nodules diagnosis was confirmed by Cervical Ultrasonography performed between 2007 and 2017 following the same diagnostic protocol during the study period. Data was obtained from medical records and interviews with the patients during routine medical care visits to assess the relationship between parameters indicative of obesity, dyslipidemia, insulin resistance, and cardiovascular risk, and thyroid nodules ultrasound characteristics as size, number, consistency, echogenicity, regularity of margins, type of vascularization on Doppler, calcification and thyroid volume; (ii) functional and autoimmune characteristics as serum concentrations of free thyroxine (FT4), thyrotropin (TSH), anti-thyroperoxidase (TPOAb) and anti-thyroglobulin (TgAb) antibodies, (iii) cytological diagnosis classified from II to VI by the Bethesda System; and (iv) pathological characteristics of follicular carcinoma or papillary carcinoma.

Regarding these parameters, the assessments considered age (in full years at the time of inclusion in the study), sex (male or female), weight (in Kg), height (in meters), Body Mass Index (BMI, kg/m^2^, classified as normal, overweight, and obese), Abdominal Circumference (measured in centimeter at the navel line, at the midpoint between the last two ribs and the upper part of the iliac bone), smoking habit (active, ex-smoker or non-smoker); diagnosis and treatment of arterial hypertension, type 2 *Diabetes Mellitus diagnosis*, and/or dyslipidemia, in addition to the serum lipid profile.

Carbohydrate metabolism was assessed using HBA1c and fasting glucose values, and cardiovascular risk, by Framingham and ASCVD (Atherosclerotic Cardiovascular Disease) scores calculated by the Guidelines On-The-Go applications, developed by the American Heart Association and ASCVD Risk Estimator Plus, developed by the American College of Cardiology, respectively. These scores estimate the 10-year risk of death from cardiovascular disease and were developed to guide the treatment and prevention of atherosclerotic cardiovascular disease in the general population.

The study was approved by the Ethics Committee for Research Involving Human Subjects of the University of Campinas with the number CAAE: 11329419.6.0000.5404. Participation in the study was conditional upon obtaining the informed consent form, which was read and signed by the participants. The study adheres to the Declaration of Helsinki: https://www.wma.net/policies-post/wma-declaration-of helsinki-ethical-principles-for-medical-research-involving human-subjects/.

### Exclusion criteria

Acutely ill patients with a cardiovascular event for less than 6 months (acute myocardial infarction, unstable angina or stroke), with a malignant neoplasm or active inflammatory disease, use of amiodarone or glucocorticoids, propranolol and/or iodinated contrast for less than 3 months before the baseline data, heart failure (New York Heart Association class III or IV), severe liver disease (decreased albumin or increased INR), advanced chronic kidney disease (glomerular filtration rate below 30 ml/h/m^2^ or hemodialysis patients).

### Cardiovascular risk assessment

The cardiovascular risk was assessed by BMI (kg/m^2^), relative fat mass (RFM) (cm/m^2^), Framingham and ASCVD scores, and the measurements of the serum total cholesterol, HDL, LDL (mg/dl), fasting glucose, glycated hemoglobin (HbA1c), and waist circumference (cm^2^).

### Carbohydrate metabolism assessment

Carbohydrate metabolism was assessed by HbA1c (high-performance liquid chromatography, HPLC, reference values RV: 4–6%) and fasting blood glucose (colorimetric enzymatic method; reference values between 70 mg/dL (3.9 mmol/L) and 100 mg/dL (5.6 mmol/L).

### Thyroid function and thyroid autoimmunity determinants

TSH was measured by electrochemiluminescence, Roche Cobas Elecsys – RV 0.41 to 4.5 μUI/mL; FT4L by Elecsys FT4 II competitive chemiluminescence immunoassay, RV 0.9 to 1.8 ng/ml; TPOAb and TgAb by Elecsys chemiluminescent immunometric test, RV up to 34UI/ml and up to 115UI/ml, respectively.

### Ultrasound criteria

The characterization of thyroid nodules was based on Regularity of the margins (regular, or irregular); Echogenicity (hypo, iso or hyperechogenic); Characteristic of the parenchyma (solid, cystic or mixed); Vascularization (central, peripheral, mixed or absent); Calcification (microcalcification, gross calcification); Value of the largest diameter of the largest nodule (mm) and Volume of the largest nodule (cm^3^). The glandular parameters were evaluated by number of nodules, Total gland volume, Presence of diving goiter (yes or no).

### Statistical analysis

Frequency tables to describe the characteristics of the sample according to the ultrasonographic, functional, cytological and anatomopathological characteristics of the thyroid nodules with the cardiovascular risk factors and parameters that make up the Metabolic Syndrome, were produced to assess the relationship between categorical variables and absolute frequency values (*n*) and percentage (%) in the column and in the row and use of Chi-square or Fisher’s exact test. To assess the relationship between categorical and numerical variables, descriptive measures (*n*, mean, standard deviation, minimum, median, and maximum) of the numerical variables were de obtained in each categorical ones, and the Mann–Whitney or Kruskal–Wallis tests were used to assess the relationship. To verify the relationship between numerical variables, Spearman’s correlation coefficients were used. The significance level adopted was 5%.

## Results

The present study included 160 patients with thyroid nodule, 136 women (85.53%) and 23 men (14.37%); 62 with obesity (41.3%), 50 overweight (33.33%); 37 with type 2 Diabetes Mellitus (24.18%), 97 with arterial hypertension (63.4%), 14 patients with a history of previous cardiovascular disease (9.15%), 58 with diagnosis of dyslipidemia (38.16%) and 19 smokers (11.95%). Of the whole patients, 59 had Papillary Thyroid Carcinoma (38.5%) and 13 had Follicular Carcinoma (8.5%). Regarding FNAB, 53 of these patients were classified as Bethesda II (34.64%), 13 as Bethesda III (8.5%), 17 as Bethesda IV (11.11%), 41 as Bethesda V (26.8%), and 13 as Bethesda VI (8.5%).

### Obesity/overweight, type 2 diabetes mellitus, cardiovascular risk, and characteristics of thyroid nodules

The presence of microcalcifications was associated with higher values of Framingham and ASCVD Cardiovascular Risk Scores, fasting glucose, and glycated hemoglobin. Ultrasonographic (US) Characteristics such as echogenicity, vascularity, nodule margins, parenchymal composition, and TIRADS were not associated with these variables (Table 1).

**Table 1.** Cardiovascular risk and ultrasonographic characteristics of thyroid nodules.

Variable			BMI	RFM	AC	Score ASCVD	Framingham score	Glycated hemoglobin (%)	Fasting blood glucose (mg/dL)
**US Echogenicity**	**Hipo**	No	28.47 ± 6.36	40.93 ± 7.82	98.25 ± 15.25	14.07 ± 12.75	12.3 ± 9.27	6.43 ± 1.65	105.39 ± 30.34
		Yes	30.26 ± 6.96	41.19 ± 5.99	99.51 ± 13.26	14.47 ± 12.1	14.35 ± 8.38	6.23 ± 1.59	100.47 ± 29.69
		***p* value**	0.1879	0.7824	0.6438	0.6568	0.1992	0.7121	0.3766
	**Hiper**	No	29.74 ± 7	40.93 ± 6.63	98.7 ± 14.06	13.62 ± 12.36	12.73 ± 9.03	6.33 ± 1.59	102.3 ± 30.79
		Yes	29.39 ± 2.56	43.53 ± 4.58	104.83 ± 8.35	21.37 ± 14.02	14.13 ± 11.26	5.38 ± 0.55	97.11 ± 9.33
		***p* value**	0.8203	0.2795	0.1672	0.1055	0.819	0.1844	0.637
	**Iso**	No	30.15 ± 6.81	40.84 ± 6.49	99.33 ± 13.23	15.94 ± 12.49	13.78 ± 9.03	6.18 ± 1.52	99.87 ± 28.58
		Yes	28.33 ± 6.76	42.18 ± 6.72	98.41 ± 16.19	13.58 ± 12.61	12.41 ± 9.11	6.61 ± 1.7	108.75 ± 33.27
		***p* value**	0.1336	0.4362	0.6556	0.2762	0.4145	0.2741	0.1963
**US Margin**	**Regular**	No	30.13 ± 6.87	41 ± 7.51	98.81 ± 17.42	17.42 ± 16.95	14.57 ± 10.42	6.33 ± 1.15	108.73 ± 40.58
		Yes	29.58 ± 6.82	41.13 ± 6.32	99.22 ± 12.92	13.17 ± 12.45	12.26 ± 8.63	6.28 ± 1.68	99.79 ± 25.37
		***p* value**	0.72203	0.9264	0.7203	0.1314	0.4602	0.3044	0.4643
	**Irregular**	No	29.82 ± 6.81	41.39 ± 6.36	99.57 ± 13	13.64 ± 12.36	12.9 ± 9.13	6.29 ± 1.64	100.16 ± 25.75
		Yes	29.3 ± 6.93	39.62 ± 7.42	96.85 ± 17.74	16.42 ± 13.5	12.29 ± 9.01	6.32 ± 1.25	109.54 ± 43
		***p* value**	0.5830	0.3162	0.3734	0.4138	0.8294	0.5389	0.5362
**US Parenchyma Characteristics**	**Solid**	No	27.74 ± 6.19	41.16 ± 7.93	99.76 ± 14.92	11.87 ± 12.3	10.17 ± 8.35	6.21 ± 1.51	100.69 ± 35.12
		Yes	30.18 ± 6.9	41.1 ± 6	98.94 ± 13.5	14.64 ± 12.63	13.44 ± 9.17	6.31 ± 1.59	102.25 ± 28.65
		***p* value**	0.1436	0.4414	0.6674	0.2928	0.1454	0.4714	0.2090
	**Mixed**	No	30.19 ± 6.85	41.27 ± 6	99.59 ± 13.62	14.68 ± 12.64	13.59 ± 9.34	6.33 ± 1.59	102.2 ± 28.4
		Yes	27.48 ± 6.29	40.6 ± 8.17	97.63 ± 14.6	11.44 ± 12.11	9.32 ± 6.91	6.16 ± 1.53	100.83 ± 36.58
		***p* value**	0.0933	0.8623	0.7	0.2822	0.0996	0.2647	0.1384
**US Vascularization**	**Peripheral**	No	30 ± 6.77	41.91 ± 5.76	100.36 ± 12.95	13.45 ± 12.9	12.61 ± 9.16)	6.25 ± 1.7	100.26 ± 25.48
		Yes	29.22 ± 6.94	40.17 ± 7.29	97.71 ± 14.76	15.18 ± 12.12	13.12 ± 9	6.36 ± 1.39	104.69 ± 35.93
		***p* value**	0.7656	0.2513	0.3277	0.5457	0.6854	0.5322	0.9839
	**Mixed**	No	29.8 ± 7.24	40.32 ± 7.05	98.46 ± 13.98	13.98 ± 12.33	11.79 ± 8.22	6.27 ± 1.59	101.39 ± 32.32
		Yes	29.64 ± 6.42	42.32 ± 5.5	100.18 ± 13.63	14.36 ± 12.95	13.79 ± 9.82	6.32 ± 1.56	102.57 ± 27.15
		***p* value**	0.9833	0.2486	0.7305	0.8291	0.4497	0.8391	0.2228
	**Central**	No	29.55 ± 6.5	41.36 ± 6.51	99.71 ± 13.81	14.23 ± 12.59	12.92 ± 9.24	6.21 ± 1.37	102.28 ± 30.27
		Yes	32.37 ± 10.8	38.86 ± 6.59	93.88 ± 13.26	12.13 ± 13.4	10.88 ± 6.09	8.03 ± 3.96	94.83 ± 19.11
		***p* value**	0.9397	0.2058	0.2345	0.8327	0.8284	0.3161	0.8159
**Microcalcification**		No	29.41 ± 7.09	40.65 ± 6.8	98.14 ± 13.25	12.58 ± 11.75	11.78 ± 8.72	5.97 ± 1.13	98.96 ± 29.84
		Yes	30.59 ± 5.97	42.81 ± 5.22	102.78 ± 15.42	19.72 ± 13.99	16.63 ± 9.54	7.24 ± 2.22	110.94 ± 28.48
		***p* value**	0.2960	0.3591	0.2986	**0.0413**	**0.0427**	**0.0013**	**0.001**
**TIRADS**		III	28.09 ± 6.17	40.64 ± 7.39	95.76 ± 13.25	11.65 ± 11.25	8.88 ± 5.74	5.88 ± 0.88	100.14 ± 26.36
		IV	30.12 ± 6.83	41.28 ± 6.47	100.22 ± 12.96	15.25 ± 13.34	13.87 ± 9.53	6.44 ± 1.77	103.57 ± 33
		V	31.19 ± 7.85	40.79 ± 6.1	99.1 ± 19.54	15.84 ± 11.9	14.61 ± 10.31	6.66 ± 1.56	100.64 ± 15.74
		***p* value**	0.3277	0.8325	0.3984	0.5288	0.2929	0.8440	0.2877

BMI = Body mass index, RFM = relative fat mass index, AC = Abdominal circumference, Framingham score and ASCVD are scores created to assess the risk of cardiovascular death over the next 10 years, TIRADS = Ultrasonographic Classification Table of Thyroid Nodules, US = Ultrasound.

Values expressed in mean ± SD.

Regarding cardiovascular risk and cytological characteristics, patients who had nodules classified by FNAB as Bethesda II had lower fasting blood glucose; as Bethesda III, higher glycated hemoglobin levels; and Bethesda IV, higher values on both cardiovascular risk scores, respectively. Regarding the Anatomopathological characteristics, the Framingham Score was lower in patients with papillary carcinoma, as well as the abdominal waist was lower in patients with follicular thyroid carcinoma (Table 2).

**Table 2.** Cardiovascular risk, cytological and anatomopathological characteristics of thyroid nodules.

Variables		BMI	RFM	CA	Score ASCVD	Framingham score	Blood glucose (%)	Fasting glycated hemoglobin (mg/dL)
BII	No	30.2 ± 7.43	40.01 ± 7.27	98.37 ± 15.76	16.21 ± 13.76	13.41 ± 9.05	106.35 ± 33.77	6.47 ± 1.79
	Yes	28.83 ± 5.44	42.05 ± 5.73	99.8 ± 12.01	11.57 ± 10.42	12 ± 9.13	94.4 ± 19.65	5.91 ± 0.87
***p* value**		0.3117	0.2039	0.5102	0.1562	0.4057	**0.0149**	0.3001
BIII	No	29.64 ± 6.87	40.79 ± 6.61	98.57 ± 13.35	13.3 ± 11.57	12.77 ± 9.14	101.42 ± 30.04	6.12 ± 1.22
	Yes	30.61 ± 6.44	44.67 ± 4.39	105.43 ± 17.86	22.53 ± 18.72	13.04 ± 8.59	108 ± 28.37	7.87 ± 3.09
***p* value**		0.6186	0.1636	0.3628	0.1658	0.7893	0.1877	**0.0316**
BIV	No	29.45 ± 6.74	41.38 ± 6.39	99.5 ± 13.8	13.38 ± 12.58	12.08 ± 8.76	101.36 ± 30.12	6.36 ± 1.65
	Yes	31.81 ± 7.29	34.08 ± 7.32	89.67 ± 11.5	21.73 ± 9.95	18.91 ± 9.82	107.62 ± 27.75	5.91 ± 0.91
***p* value**		0.1468	0.0832	0.2316	**0.0357**	**0.0472**	0.1217	0.4266
BV	No	29.85 ± 6.58	41.21 ± 6.55	99.04 ± 13.78	14.19 ± 12.1	12.99 ± 9.14	99.2 ± 24.79	6.29 ± 1.62
	Yes	29.36 ± 7.53	39.9 ± 6.51	100.5 ± 15.06	14.05 ± 14.27	12.14 ± 8.96	109.61 ± 40.23	6.3 ± 1.46
***p* value**		0.1362	0.8507	0.9333	0.6986	0.7443	0.2021	0.982
BVI	No	29.52 ± 6.14	41.31 ± 6.54	99.53 ± 13.46	14.49 ± 12.63	12.86 ± 9.16	101.64 ± 30.48	6.27 ± 1.6
	Yes	30.11 ± 8.04	39.66 ± 6.49	96.3 ± 16.48	5 ± 4.88	11.70 ± 8.02	105.25 ± 23.25	6.7 ± 0.85
***p* value**		0.8004	0.3959	0.8342	0.1721	0.8207	0.2843	0.1048
Papillary carcinoma	No	29.49 ± 7.26	36.01 ± 7.28	91.5 ± 10.34	22.3 ± 11.03	13.34 ± 4.45	112.36 ± 30.74	6.14 ± 1.06
	Yes	31.15 ± 7.45	40.97 ± 5.61	102 ± 12.06	10.62 ± 13.09	10.61 ± 8.2	105.2 ± 36.81	6.35 ± 2.15
***p* value**		0.4062	0.2932	0.1760	0.2247	**0.0323**	0.2053	0.7714
Follicular carcinoma	No	30.94 ± 7.19	40.42 ± 6.83	103.78 ± 8.41	14.67 ± 11.52	13.78 ± 9.03	105.96 ± 36.05	6.32 ± 2.02
	Yes	28.74 ± 9.04	36 ± 3.74	82.67 ± 5.69	13.58 ± 12.61	12.41 ± 9.11	116 ± 29.39	6.13 ± 0.88
***p* value**		0.2979	0.2294	**0.0124**	0.2762	0.4145	0.434	0.6349

Values expressed in mean ± SD.

BMI = body mass index, RFM = relative fat mass index, AC = Abdominal circumference, Framingham Score and ASCVD are scores created to assess the risk of cardiovascular death over the next 10 years, BII = Bethesda II = benign nodule, BIII = Bethesda III = atypia or follicular lesion of undetermined significance, BIV = Bethesda IV = Suspected for follicular neoplasm, BV = Bethesda V = Suspected of malignancy, BVI = Bethesda VI = malignant.

The presence or absence of thyroid autoimmunity did not show a significant association with BMI, RFM, Abdominal Circumference, Cardiovascular Risk Scores, diagnosis of type 2 diabetes mellitus, and dyslipidemia (Table 3).

**Table 3.** Cardiovascular risk factors and presence or absence of thyroid antibodies.

Variable		BMI	RFM	AC	ASCVD Score	Framingham Score	Type 2 Diabetes Mellitus		Dyslipidemia	
		Mean ± SD	Mean ± SD	Mean ± SD	Mean ± SD	Mean ± SD	Yes	No	Yes	No
TgAb	Yes	27.18 ± 7.84	38.61 ± 4.41	96.83 ± 15.96	9.21 ± 11.03	12.05 ± 8.05	1 (4.17%)	16 (19.51%)	2 (6.06%)	15 (20.83%)
	No	30.11 ± 6.49	41.93 ± 6.42	101.3 ± 13.51	13.15 ± 12.58	11.47 ± 8.54	23 (95.83%)	66 (80.49%)	31 (93.94%)	57 (79.17%)
***p* value**		0.0883	0.0847	0.2065	0.3544	0.685	0.1114		0.0564	
TPOAb	Yes	28.29 ± 5.97	41.82 ± 3.99	95.56 ± 9.66	12.43 ± 12.6	11.84 ± 8.55	2 (8%)	16 (18.18%)	3 (8.33%)	15 (19.74%)
	No	29.59 ± 6.62	41.22 ± 7.04	101.15 ± 14.49	12.74 ± 12.22	11.51 ± 8.33	23 (92%)	72 (81.82%)	33 (91.67%)	61 (80.26%)
***p* value**		0.8004	0.3959	0.8342	0.9836	0.7832	0.3534		0.1249	

BMI = Body mass index, RFM = Relative fat mass index, AC = Abdominal Circumference, Framingham Score and ASCVD are scores created to assess the risk of cardiovascular death over the next 10 years, Actg = Anti-thyroglobulin antibody, ACTPO = Anti-thyroperoxidase antibody, SD = Standard deviation, Ƿ = Spearman coefficient correlation, BMI = Body mass index, RFM = Relative fat mass index, AC = Abdominal circumference, Framingham Score and ASCVD scores: to assess the risk of cardiovascular death over the next 10 years, FT4 = Free thyroxine hormone, TSH = Thyrostimulating hormone.

Regarding correlations between cardiovascular risk factors, thyroid function, and ultrasound characteristics of thyroid nodules, the following correlations were showed: gland volume was positively correlated with RFM, waist circumference, fasting blood glucose, and glycated hemoglobin. The number of nodules was directly correlated with BMI and glycated hemoglobin values. The diameter of the largest nodule was directly correlated with values of waist circumference, fasting blood glucose, and glycated hemoglobin. The volume of the largest nodule was directly correlated with the waist circumference and glycated hemoglobin values. FT4 was directly correlated with fasting blood glucose and ASCVD scores. TSH were negatively correlated with RFM (Table 4).

**Table 4.** Correlations between cardiovascular risk factors and thyroid function and thyroid nodule characteristics.

Variable	FT4		TSH		Gland volume		Nodules number		Largest Nodule diameter		Nodule volume	
	Ƿ/*p* valor											
BMI	0.04188	0.612	−0.09134	0.2679	0.09857	0.2536	***0.17044***	***0.0377***	0.1141	0.1703	0.07387	0.3755
RFM	−0.08157	0.4635	***−0.32067***	***0.0031***	***0.30312***	***0.0091***	0.19144	0.083	0.20377	0.0681	0.16649	0.1374
AC	−0.1378	0.2141	−0.20726	0.0601	***0.27462***	***0.0187***	0.16413	0.1382	***0.30394***	***0.0058***	***0.24993***	***0.0244***
Fasting blood glucose	0.17190	0.0454	0.06334	0.4638	0.21339	0.0169	0.05750	0.5061	***0.19663***	***0.0228***	0.16214	0.0603
Glycated hemoglobin	−0.00914	0.9315	0.13981	0.1862	0.24290	0.0269	0.21406	0.0416	***0.27536***	***0.0086***	***0.22437***	***0.0335***
ASCVD score	***0.27893***	***0.0093***	0.11267	0.3017	0.17346	0.1288	−0.01551	0.8873	−0.03463	0.7545	−0.05922	0.5926
Framingham score	0.14274	0.1653	0.17995	0.0794	0.04789	0.654	0.05147	0.6185	0.04884	0.6402	0.01173	0.9107

Ƿ = Spearman coefficient correlation, BMI = body mass index, RFM = relative fat mass index, AC = Abdominal circumference, Framingham Score and ASCVD scores: to assess the risk of cardiovascular death over the next 10 years, FT4 = free thyroxine hormone, TSH = thyrostimulating hormone.

### Associated factors to cytological diagnosis of malignancy (Bethesda cytology V and VI)

Univariate logistic regression analysis showed that higher FT4 concentrations increased the chance of malignant cytology by 4.7 times. Similarly, for every unit of HDL that increased, there was an increase in the chances of malignancy by 3%. Patients who were not statin users had a 3-fold most chance of malignant cytology (OR = 1/0.316 = 3.1645). In multivariate logistic regression analysis, higher HDL levels were shown to be an associated factor for malignant cytology, for every unit of HDL that increased, there was an increase in the chances of malignancy by 6.4% (OR = 1.064) (Table 5).

**Table 5.** Associated factors of malignant cytological.

Variable	*N*	Odds ratio	Confident limits 95%	*p* value
Univariate regression analysis				
Male sex	153	1.556	0.632 − 3.835	0.3363
Age at diagnosis (years)	148	0.993	0.971 − 1.015	0.5139
FT4 (ng/dl)	153	4.756	1.822 − 12.418	0.0014
TSH (mUI/L)	153	1.050	0.988–1.115	0.1146
BMI (kg/m^2^)	149	1.013	0.964–1.064	0.6204
RFM (cm/m^2^)	83	0.964	0.877–1.060	0.4526
Abdominal circumference (cm)	83	0.982	0.933–1.033	0.4855
Fasting blood glucose (mg/dl)	136	1.011	0.999–1.024	0.0668
Glycated hemoglobin (%)	91	1.044	0.792–1.376	0.7600
Treatment of T2DM with metformin	153	0.980	0.443–2.168	0.9600
Treatment of T2DM with insulin	153	0.942	0.167–5.316	0.9457
Dyslipidemia	152	0.537	0.262–1.103	0.0905
Treatment of dyslipidemia with statin	153	0.316	0.134–0.744	0.0083
LDL (mg/dl)	116	1.003	0.991–1.015	0.6047
HDL (mg/dl)	118	1.030	1.002–1.059	0.0389
Framingham score (%)	96	0.987	0.939–1.037	0.6037
ASCVD score (%)	86	0.988	0.950–1.028	0.5630
Multivariate regression analysis				
HDL (mg/dl)	48	1.064	1.012–1.119	0.0158

Multivariate regression analysis (Model without RFM and CA), FT4 = Free thyroxine, TSH = Thyroid stimulating hormone, BMI = Body mass index, RFM = Relative fat mass, LDL = Low density lipoprotein, HDL = High density lipoprotein.

### Associated factors of differentiated thyroid carcinoma

Univariate logistic regression analysis showed a highest odds ratio of differentiated thyroid carcinoma (papillary microcarcinoma + papillary carcinoma + follicular carcinoma) and younger patients (OR = 1/0.967 = 1,034); furthermore, higher serum FT4 increased this chance in 10-fold. Additionally, higher serum FT4 concentrations were shown as an associated factor for Differentiated Thyroid Carcinoma (OR = 7.409) in the multivariate logistic regression analysis (Table 6).

**Table 6.** Factors associated with the diagnosis of differentiated thyroid carcinoma.

Variables	*N*	Odds ratio	Confident limits 95%	*p* value
Univariate regression analysis				
Male sex	159	1.344	0.540–3.343	0.5246
Age at diagnosis (years)	153	0.967	0.945–0.989	0.0041
FT4 (ng/dl)	153	10.725	3.846–32.990	<0.0001
TSH (mUI/L)	153	1.010	0.964–1.058	0.6892
BMI (kg/m^2^)	149	1.035	0.985–1.087	0.1771
RFM (cm/m^2^)	83	0.972	0.886–1.066	0.5440
Abdominal circumference (cm)	88	0.996	0.952–1.042	0.8619
Blood glucose (mg/dl)	136	1.007	0.995–1.019	0.2303
Glycated hemoglobin (%)	91	1.103	0.842–1.446	0.4765
Metformin use	153	0.980	0.443–2.168	0.9600
Insulin use	153	0.365	0.042–3.211	0.3639
Dyslipidemia	152	0.794	0.395–1.595	0.5171
Statin use	153	0.622	0.288–1.341	0.2258
LDL (mg/dl)	116	1.003	0.991–1.016	0.6214
HDL (mg/dl)	118	1.024	0.996–1.054	0.0919
Framingham score (%)	96	0.988	0.939–1.039	0.6278
ASCVD score (%)	86	0.973	0.930–1.018	0.2335
Multivariate regression analysis				
FT4 (ng/dl)	63	7.409	1.681–32.653	**0.0081**

Multivariate regression analysis (model without RFM, CA and ASCVD score).

## Discussion

This study verified that higher serum free T4 concentrations, within reference values and in euthyroid patients, increased the chance of malignant cytology by 4.7 times and evidenced to be an associated factor that increased by 7-fold the chance of Differentiated Thyroid Carcinoma. These findings have been described in the literature *in vitro* and in animal models studies, demonstrating possible effects of T3 and T4 hormones on cell proliferation and apoptosis, and on cancer angiogenesis process [6]. In addition, the association between higher concentrations of FT4 and the development of various types of cancer is widely cited as breast, prostate, and lung cancer, however, there are few studies specifically relate it to thyroid cancer [7]. In this sense, Kim et al. in a large cohort study, showed that low-normal TSH and high-normal FT4 levels in euthyroid individuals were linked to a higher thyroid cancer risk [8]. This finding could support the biological plausibility of FT4 as a predictor of malignancy and highlights the need to reassess the clinical meaning of FT4 levels considered as in a normal range.

Additionally, this study showed a 6.4% higher chance of malignant cytology for each unit of elevation in HDL level, identifying the HDL as an associated factor for Bethesda V and VI categories. However, HDL was not associated with patients with confirmed diagnosis of thyroid cancer, raising discussion about the influence of lipids on thyroid malignancy. Zhang et al. [9] found that Bethesda category, TIRADS, nodule size, and serum levels of HDL-C were most significant differentiators for patients with PTC and those with benign nodules. On the other hand, some authors found that in patients with metabolic syndrome, HDL-C may have a protective effect against thyroid cancer, since obesity, insulin resistance, hyperglycemia, and aberrant lipid metabolism act separately or together in multiple physiological pathways to promote the development and progression of cancer [10]. Additionally, low levels of triglyceride and HDL were independently associated with the recurrence of PTC [11]. Furthermore, Liam and Sun [12] in a Mendelian randomization study provided evidence that metabolic factors like obesity, levels of HDL and triglycerides, as well as insulin resistance could causally contribute to thyroid cancer risk, addressing a limitation related to the cross-sectional and observational design of the present study, which prevents causal inference.

Regarding the ultrasonographic characteristics of the thyroid nodules, the presence of microcalcification was associated with higher values of the Framingham and ASCVD Cardiovascular Risk Scores, elevated levels of fasting blood glucose and higher glycated hemoglobin, suggesting a relationship between factors associated with RCV and glycemic alterations and nodules with greater malignancy potential, as verified that microcalcification is one of the most specific ultrasound features for malignancy. Consistent with this finding, a large study [13] showed a 2.67-fold increase in the risk of microcalcification in men with Metabolic Syndrome. Furthermore, meta-analysis showed that insulin resistance, dysglycemia, higher BMI values and arterial hypertension are significantly associated with a higher risk of developing thyroid cancer [14].

Still on the ultrasonographic characteristics, the greater volume of the gland was correlated with greater FMR, waist circumference, blood fasting glucose, and glycated hemoglobin. Furthermore, the greater number of nodules showed correlation with greater values of BMI and glycated hemoglobin. Additionally, greater nodular volume was associated with greater waist circumference and highest glycated hemoglobin. Such findings suggest that larger goiters and thyroid nodules, as well as greater nodularity were related to factors indicative of insulin resistance and could be explained by the stimulus exerted by insulin in cell replication and anti-apoptotic effects, leading to tissue proliferation and hyperplasia [15].

Concerning cytological characteristics, patients who had nodules classified by FNAB as Bethesda II had lower fasting blood glucose. On the other hand, Bethesda III nodules were associated with higher glycated hemoglobin, and Bethesda IV nodules, at higher values in both cardiovascular risk scores, corroborating previous findings and suggesting that benign lesions would not be related to higher cardiovascular risk or changes in insulin action.

About the anatomopathological features, the Framingham Score was lower in patients with papillary carcinoma, as well as the abdominal waist was lower in patients with follicular thyroid carcinoma. The scientific literature is uncertain whether greater measure of abdominal circumference would be associated with a higher risk of thyroid cancer; many studies have shown no relationship between obesity and this type of cancer or reduced risk of cancer in obese patients [16,17].

Interestingly, the presence of thyroid autoimmunity did not influence the factors indicative of cardiovascular risk or insulin resistance evaluated.

This study has as strengths the fact that all anthropometric parameters and cardiovascular risk assessments were performed by the same researcher, observing quality control and metrics. Additionally, the pathological examination was used as the gold standard method to prove the malignancy of thyroid nodules. A limitation is the relatively small sample size, mainly to perform the multivariate analysis that might compromise the statistical power. Additionally, the type of epidemiological study, observational and cross-sectional, does not allow establishing causality, although there is evidence of causality between metabolic factors and risk of thyroid cancer.

In conclusion, thyroxine in higher concentrations in euthyroid patients was shown to be a factor associated with malignant cytology and Differentiated Thyroid Carcinoma. Nodules with higher malignancy potential on ultrasound were associated with greater cardiovascular risk and glucose metabolism alteration, unlike what was found in benign lesions. Larger and multiple goiters and nodules were related to factors indicative of insulin resistance. Furthermore, higher HDL and non-use of statin were associated with malignant cytology, contrary to what was found in patients using these drugs, who exhibited a lower chance of malignancy. Based on our findings, there is a potential clinical application regarding the concomitant assessment of cardiovascular risk (including lipid and glycemic profile), as well as factors indicative of insulin resistance, aiming to contribute to the investigation of malignancy in patients with thyroid nodules.

Thus, malignant, and potentially malignant nodules seem to be related to hormonal factors that induce greater cell replication, such as thyroxine and insulin resistance and alterations in glucose metabolism. However, these findings need further studies to be properly confirmed.

## Data Availability

We agree to make the data and materials supporting the findings or analysis in our article available upon reasonable request. The readers can contact DEZW for the data request.
